# Graduate Institutions in London

**Published:** 1918-09-07

**Authors:** 


### GRADUATE INSTITUTIONS IN LONDON.

**London School of Clinical Medicine (Post-Graduate).**—This School, which is devoted exclusively to post-graduate study, has its headquarters at the "Dreadnought" Hospital, Greenwich. Affiliated to it for the purposes of teaching are the following institutions :—The Royal Waterloo Hospital for Children and Women, the Bethlem Royal Hospital, and the Miller General Hospital. At the "Dreadnought" Hospital there are in addition to the medical and surgical wards special wards for the treatment of patients suffering from venereal disease, the medical and surgical forms of tuberculosis, diseases of the eye, ear, nose, and throat. In addition there are unusual opportunities for practical instruction in operative surgery and pathology. Every variety of disease may be studied at the "Dreadnought" and the hospitals affiliated thereto. In consequence of the war the School has been temporarily closed.

The war has seriously affected this School—so much so that the entire ordinary curriculum has been abandoned. This was rendered necessary in consequence of the departure, in connection with the war, of a large number of members of the teaching staff. The only portion of the School which remains open is that which affords facilities for instruction in Operative Surgery. There is an ample supply of material, and a good many graduates have attended the course, some of these being American and foreign graduates. The pathological examinations and work in the laboratories will be carried on under Professor R. Tanner Hewlett, the Director of Pathology. Dr. H. Ridley Prentice, a member of the honorary medical staff, has generously given his services as medical superintendent in the hospital. Women students are not admitted to the practice of the Hospital or to the lectures in the School.

A syllabus of the course of study which takes place at ordinary times may be obtained on application to the Secretary, P. Michelli, C.M.G., "Dreadnought" Hospital, Greenwich.

**North-East London Post-Graduate College.**—This School, which is in connection with the Prince of Wales's General Hospital, Tottenham, N., is recognised by the University of London, the Admiralty, and the India Office, and by the University of Cambridge in regard to bacteriology for the D.P.H. diploma, as a place of post-graduate study. Opportunities are here afforded for attending demonstrations on various branches of medicine, surgery, and gynæcology, diseases of the eye, ear, throat, nose, skin, fevers, diseases of children, psychological medicine, anæsthetics, *x*-rays, bacteriology, and dentistry. Special classes with a limited attendance have been arranged for these and other subjects, but they are largely in abeyance for the present owing to the war. The fee for a three-months' course in any single department is one guinea, or three guineas admits for a similar term to the whole practice of the hospital. A perpetual ticket costs ten guineas. Owing to exigencies arising out of the war there will be no formal opening lecture in the ensuing session, but the full work of the hospital will commence at the beginning of October. Additional information from the Dean.

**West London Post-Graduate College.**—The West London Hospital, Hammersmith, was the first of the general hospitals in London to reserve its practice strictly for qualified men, and to establish a post-graduate College as an integral part of its constitution. The hospital itself contains 160 beds, and the advantages of the direct connection of the College with the wards and out-patient work are obvious. It is recognised by the Royal College of Surgeons as an institution where candidates (who need not be members) for the Fellowship can spend the necessary year of study after obtaining a registrable qualification, while the College and hospital are also recognised by the naval and military authorities as regards study leave. The hospital is authorised by the University of London to give certificates of post-graduate study for the M.D. and M.S. degrees. Members of the College may also accompany the resident staff on their visit to the wards. Operations are performed daily at 2.30 P.M. Post-graduates are allowed to stand near the table and can see the operations perfectly. The surgeons often avail themselves of the assistance of post-graduates. The out-patient departments necessarily resemble in their main features those of other general hospitals. During the sessions lectures are given daily, except Saturdays, at 5 P.M., on practical medicine and surgery in all their branches. There are also short courses on tropical and mental diseases by specialists in those subjects.

Small classes are formed, when required, in connection with diseases of special regions, such as eye, throat, skin, etc.; for the clinical examination of blood and urine; in vaccine therapy, bacteriology and medical microscopy generally; in applied anatomy, administration of anæsthetics, gastro-intestinal surgery, cystoscopy, *x*-ray work, etc. For these classes, each of which is strictly limited to a small number, additional fees are required, as they are practically individual and tutorial. The ordinary fee for membership covers attendance at all general lectures and at all the routine practice of the hospital, whether in the general or special wards or departments. Autopsies can, of course, be attended. Arrangements can be made through the College authorities for special coaching should it be desired. The V.D. Department is open daily, Saturdays included, at 5.30 P.M.

Members may join for any period from one week upwards, the fees ranging from one guinea to £30.

